# Feasibility Test of a Liquid Film Thickness Sensor on a Flexible Printed Circuit Board Using a Three-Electrode Conductance Method

**DOI:** 10.3390/s17010042

**Published:** 2016-12-27

**Authors:** Kyu Byung Lee, Jong Rok Kim, Goon Cherl Park, Hyoung Kyu Cho

**Affiliations:** 1 Department of Nuclear Engineering, Seoul National University, Seoul 08826, Korea; kblee@kins.re.kr (K.B.L.); parkgc@snu.ac.kr (G.C.P.); 2 Department of Nuclear Safety, Korea Institute of Nuclear Safety, Daejeon 34142, Korea; 3 Thermal Hydraulics Safety Research Division, Korea Atomic Energy Research Institute, Daejeon 34057, Korea; jongrok@kaeri.re.kr

**Keywords:** liquid film thickness, liquid film sensor, three electrode conductance method, two-phase flow experiment

## Abstract

Liquid film thickness measurements under temperature-varying conditions in a two-phase flow are of great importance to refining our understanding of two-phase flows. In order to overcome the limitations of the conventional electrical means of measuring the thickness of a liquid film, this study proposes a three-electrode conductance method, with the device fabricated on a flexible printed circuit board (FPCB). The three-electrode conductance method offers the advantage of applicability under conditions with varying temperatures in principle, while the FPCB has the advantage of usability on curved surfaces and in relatively high-temperature conditions in comparison with sensors based on a printed circuit board (PCB). Two types of prototype sensors were fabricated on an FPCB and the feasibility of both was confirmed in a calibration test conducted at different temperatures. With the calibrated sensor, liquid film thickness measurements were conducted via a falling liquid film flow experiment, and the working performance was tested.

## 1. Introduction

Understanding the characteristics of liquid films in gas-liquid two-phase flows is an important part of the safety and performance analyses of nuclear power plant systems. For this reason, numerous studies have been conducted in an effort to measure local liquid film thicknesses under two-phase flow conditions. There are various methods that can be used to measure film thicknesses. Several widely applied methods are the ultrasonic, optical, neutron and electrical methods. The ultrasonic method measures the liquid film thickness using the time difference between signals reflected from the boundary interface of the medium. As the velocity of sound is affected by its medium, however, the ultrasonic method has rather low precision [1]. Therefore, this method is limited when used to measure the thicknesses of thin films. Furthermore, multiple-point measurements with the ultrasonic method are confined due to the high cost of the device [1]. Optical methods require a high-speed camera, diffraction tools, and X-ray tomography. Though most optical methods have high spatial and time resolutions, they are limited when applied to complicated flow conditions, as the light is distracted at the boundary interface [2]. Neutron-based methods usually measure the film thickness with a tomography-based approach. Though the spatial resolution of this method is sufficiently high, only time-averaged data is provided. This method also incurs high costs when setting up the measurement device. The electrical method is widely used in two-phase experiments given that it can measure not only the liquid film thickness but the void fraction. Most electrical methods use the electric conductivity of the liquid to measure the liquid film thickness. For non-conductive liquids, the capacitance method is applied to measure the film thickness. Generally, electrical methods for liquid film measurements have high time resolutions due to their electrical characteristics. However, the electrode size and geometry cause relatively low spatial resolutions in multiple-point measurements, representing a limitation of the electrical methods.

Damsohn et al. [3] proposed an electrical method on a printed circuit board (PCB) in order to measure local liquid film thicknesses and to overcome the above-mentioned limitation of the electrical method regarding the spatial resolution. A PCB, which allows the elaborate fabrication of electrodes, enabled high spatial resolutions of the liquid film thickness field to be realized. Furthermore, by coupling the sensor with a wire-mesh circuitry system, high time resolutions are achieved simultaneously. The wire-mesh sensor principle is based on a matrix-like arrangement of the measuring points. Two sets of wire electrodes are stretched perpendicularly to each other, with a small axial separation distance between them. The transmitter electrodes are sequentially activated while all receiver electrodes are parallel-sampled in such a way that the electrical conductivity of the fluid at each crossing point can be evaluated. Based on these measurements, the sensor is thus able to determine the instantaneous fluid distribution across the cross-section [3].

In other work, the electrode used in the electrical method also was fabricated on a flexible printed circuit board (FPCB) for application onto a curved surface. Arai et al. [4] and D’Aleo et al. [5] also used this method to measure the liquid film thickness in an annulus channel and in a microchannel, respectively. Coupling a PCB or FPCB with wire-mesh circuitry provides high time and space resolutions simultaneously, but the experimental condition is restricted to an isothermal condition, as the electrical conductivity of the liquid is affected by its temperature. According to Hayashi [6], the conductivity of water increases by approximately 2% when the water temperature increases by 1 °C.

The objective of this study is to develop the concept of a liquid film sensor fabricated on an FPCB, which works accurately under temperature-varying flow conditions. The motivation behind this arose from an experiment by Yang et al. [7] on the two-dimensional liquid film behavior. They measured reductions in the liquid film thickness after impingement on a rectangular duct with a transverse air flow using the ultrasonic method; the experimental data were then used to improve the wall and interfacial friction factor models of the nuclear reactor safety analysis code. However, under actual flow conditions, the working fluids are saturated steam and subcooled water. Therefore, condensation occurs at the interaction between the two phases. The condensation increases the water temperature and changes the electrical conductivity of the fluid. As a result, the conventional electrical method cannot be applied under this flow condition. Furthermore, the plate duct in their experiment simulated the unfolding downcomer annulus of a nuclear reactor pressure vessel. However, the geometry of interest in practical situations is the annulus channel rather than the plate duct. Therefore, a liquid film sensor which can be used with a curved geometry is required in order to extend the findings of Yang et al. to more realistic flow conditions. The fabrication of a liquid film sensor on an FPCB was selected for this reason.

For liquid film measurements under varying conductivity conditions, a three-electrode method was proposed initially by Coney [8] using parallel rectangular electrodes. According to him, if the receiving electrode is segmented into two parts perpendicularly to the current direction, the liquid film thickness can be measured with the current ratio acquired by the two segmented receiving electrodes. This works because the current flowing to the farther and closer parts of the segment will be proportional to the conductivity, and their ratio will depend only upon the film thickness. Kim et al. [9] conducted experimental research on the three-electrode conductance method for liquid film measurements under varying temperature conditions. They fabricated a sensor on a PCB and assessed the temperature compensation capability of the sensor.

In the present study, the three-electrode conductance method for liquid film thickness measurements by Kim et al. [9] was extended to apply the sensor to an FPCB and increase the degree of integration of the measurement points. To do this, two types of liquid film sensors were fabricated on an FPCB, one with the design from Kim et al. [9] and the other with a new design of electrode arrangement for denser integration of the measurement points. Prototype sensors were fabricated on an FPCB after sensitivity studies of the sensor performance with the electrode geometry via an electrical potential analysis. The prototype sensors were calibrated at different temperatures and the feasibility of temperature compensation was investigated. The calibrated sensor was applied in a falling liquid film experiment in varying temperature conditions, and its working performance was tested. This paper presents the procedure of the design of the sensor, the calibration results, and the feasibility test results and discusses required future improvements.

## 2. Design and Fabrication of the Prototype Sensor

### 2.1. Fabrication of the Sensor on an FPCB

**Figure 1:**
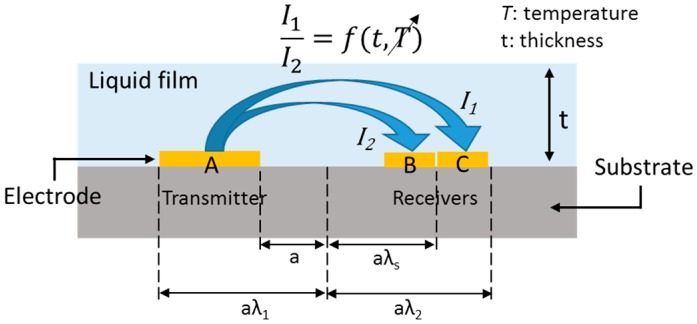
Principle of the three-electrode conductance method [9].

The three-electrode method uses the current ratio ($I_{1}/I_{2}$) to measure the film thickness, as presented in Figure 1, rather than the absolute value of the current. Coney [8] proposed the concept of the three-electrode method with conformal transformation as follows. In this theory, the current ratio can be determined by Equation (1).

(1) $$\frac{I_{1}}{I_{2}}=\frac{F\left(\beta ,m_{1}\right)}{F\left(\pi /2,m_{1}\right)-F\left(\beta ,m_{1}\right)},$$

where F(β,m_1_) is an incomplete elliptic integral of the first kind defined by Equation (2),

(2) $$F\left(\beta ,m_{1}\right)=\int_{0}^{\beta }{\left(1-m_{1}sin^{2}\alpha \right)}^{-0.5}d\alpha ,$$

where β and m_1_ are factors defined by Equations (3) and (4):

(3) $$sin^{2}\beta =\frac{sinh\frac{\pi }{2h}\left(\lambda_{2}-1\right)sinh\frac{\pi }{2h}\left(\lambda_{1}-\lambda_{s}\right)}{sinh\frac{\pi }{2h}\left(\lambda_{1}-1\right)sinh\frac{\pi }{2h}\left(\lambda_{1}+\lambda_{s}\right)},$$

(4) $$m_{1}=\frac{sinh\frac{\pi }{2h}\left(\lambda_{2}-1\right)sinh\frac{\pi }{2h}\left(\lambda_{1}-1\right)}{sinh\frac{\pi }{2h}\left(\lambda_{2}+1\right)sinh\frac{\pi }{2h}\left(\lambda_{1}+1\right)},$$

where π is a pi (3.14159…), λ_s_ and *h* are the dimensionless distance and dimensionless film thickness, respectively, and both are normalized by a. Following the equations above, the current ratio is independent of temperature changes [8]. However, the equations above were derived from simplified geometry. Therefore, electrical potential analysis was conducted for precise analysis in the present study. Because the current ratio can compensate for conductivity or temperature changes, the measurement error is expected to be minimized under a temperature-varying condition. The theoretical background of the three-ring method assumes that the impedance of the liquid film has some resistance and negligible reactance. Thus, the impedance of the liquid film is a function of the temperature and the thickness. Given that the electric conductivity is a function of the temperature, an impedance ratio of A:B and A:C only depends on the film thickness if the liquid temperature in the A–B region is identical to that in the A–C region.

**Figure 2:**
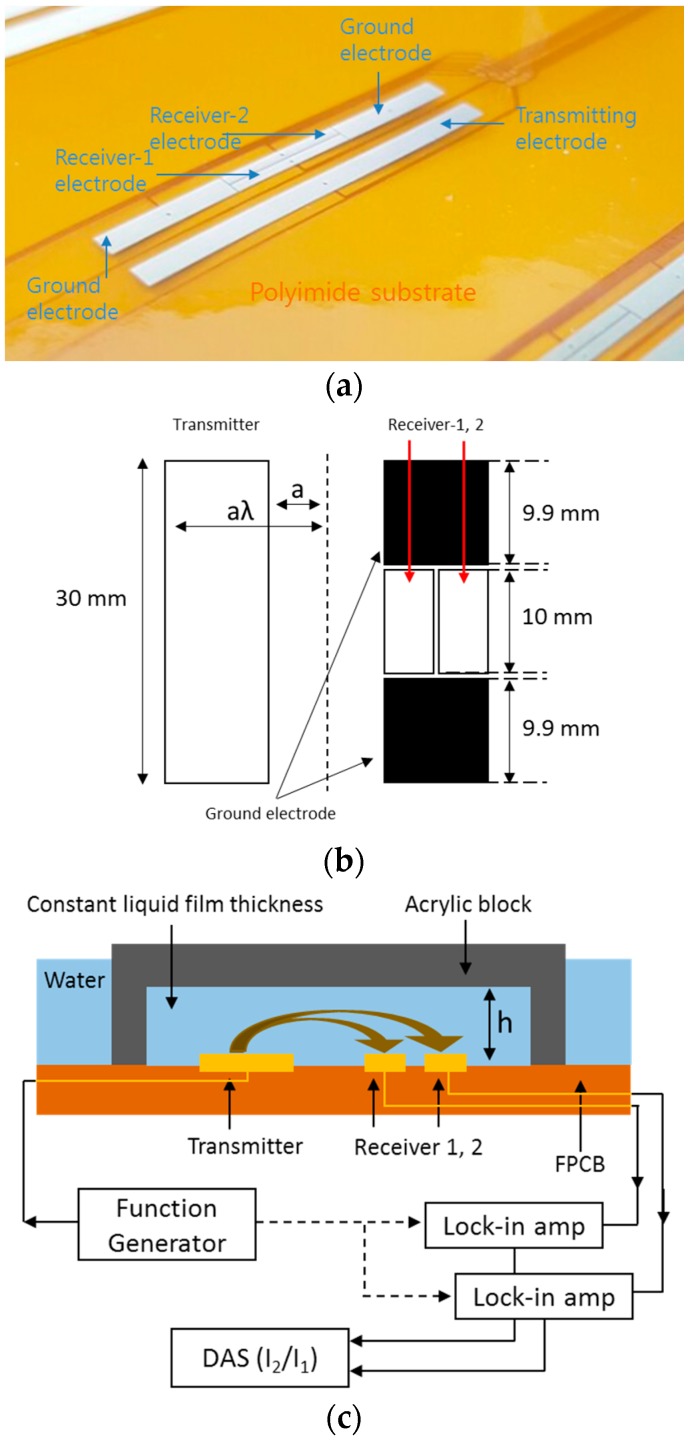
(**a**) configuration of the electrodes; (**b**) specific dimensions of the electrodes; and (**c**) calibration experiment and circuitry setup.

Based on the design proposed by Kim et al. [9], the electrodes were fabricated on an FPCB in the present study, as shown in Figure 2a. The specific dimensions of the electrodes are described in Figure 2b. The transmitter electrode is located on one side and is rectangular in shape, and the receiver-1 and receiver-2 electrodes are placed on the other side with a 0.1 mm gap between them. A ground electrode exists on both the top and bottom of the receivers to prevent an end effect, leaving a 0.1 mm gap between the receivers and the ground electrode in each case. The end effect refers to a bypass current generated around the edge of the electrode. In order to confirm the geometry effect, two electrodes of different sizes (a = 1.0 mm, λ = 3, a = 1.0 mm, and λ = 5) were manufactured.

**Figure 3:**
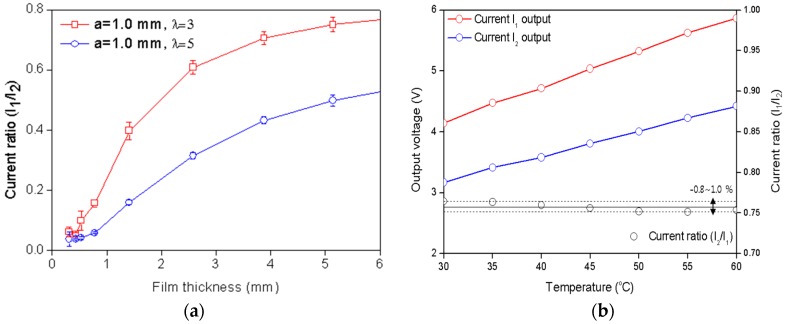
(**a**) current ratio with changes in the film thickness and electrode geometry; and (**b**) current output signal and current ratio at varying temperatures.

As shown in Figure 2c, calibration was carried out using insulated acrylic blocks, which can create different liquid film thicknesses (h = 0.3, 0.4, 0.5, 0.8, 1.4, 2.5, 3.8, and 5.1 mm). The liquid film thickness is held constant because the acrylic block confines the liquid film thickness structurally. An AC voltage signal of 0.1 V is induced from the function generator to the transmitter at a frequency of 1 kHz, and the current signals generated by the receivers are transmitted to a DAS (NI-9205, 16-bit) through a lock-in amp. Figure 3 presents the acquired experimental data with various film thicknesses of 0.5 mm–6 mm and water temperatures of 30 °C–60 °C. The increase in the current ratio with the liquid film thickness is shown in Figure 3a. When the liquid film thickness was 2.6 mm, the absolute values of the current outputs from the two receiving electrodes and their ratio with various water temperatures were compared, as shown in Figure 3b. The absolute values of the current output increase with the water temperature as the water conductivity increases. As the water temperature was increased from 30 °C to 60 °C, the output signals of $I_{1}$ and $I_{2}$ increased by 47% and 45%, respectively. However, the current ratio remained at a constant value of 0.757 within ±1.0% of error. This calibration result clearly demonstrates the advantages of the three-electrode method and confirms the feasibility of fabricating the sensor on an FPCB for liquid film thickness measurements.

### 2.2. Design of a Prototypic Sensor for Higher Integration of the Measurement Points

**Figure 4:**
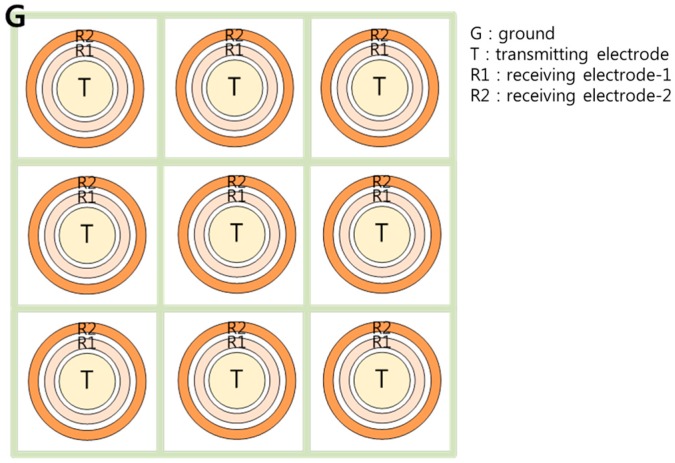
Disposition of multiple probes.

The electrode design by Kim et al. [9] in the three-electrode method presents a limitation when fabricating an integrated electrode configuration to achieve a high spatial resolution with several measurement points owing to the crosstalk effect. If the sensor elements are too close to each other, the transmitting signal can reach the neighboring sensor elements’ receiving electrodes, lowering the measurement accuracy when this occurs. This motivated the design of a new type of electrode configuration that can achieve a higher integration degree and a high spatial resolution. In the present study, a three-electrode sensor with a ring-type electrode arrangement is proposed, as shown in Figure 4, indicating a part (3 × 3 array) of the whole sensor.

The transmitter electrode is located in the center of a concentric circle, and receiver-1 and receiver-2 are positioned and enclosed within the transmitter. Given the enclosed geometry, the ring-type arrangement can prevent the end effect. The ground electrode is positioned between each sensor element to minimize the crosstalk effect. The electric current flow at each sensor element has a radial symmetric pattern such that it is easy to arrange multiple sensors, as presented in Figure 4. Thus, this configuration is advantageous for achieving higher spatial resolutions in liquid film thickness field measurements as compared to previous sensor configurations.

In order to design and optimize the geometry of the newly proposed sensor electrodes, an electrical potential field simulation was conducted using the COMSOL Multiphysics code [10]. The electrical potential field developed in a liquid film can be analyzed by solving the Maxwell equation. In order to confirm the relationship between the electrode geometry and the current ratio, an electrical potential field simulation was conducted while changing the radius of the electrode. The target measurement thickness was 0.5–3.0 mm, which is in the range used in the experiment by Yang et al. [7].

**Figure 5:**
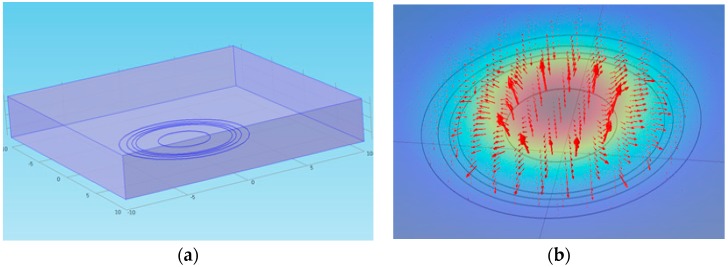
(**a**) Calculation domain of the electrical potential analysis; and (**b**) electrical potential field around the electrodes.

**Figure 6:**
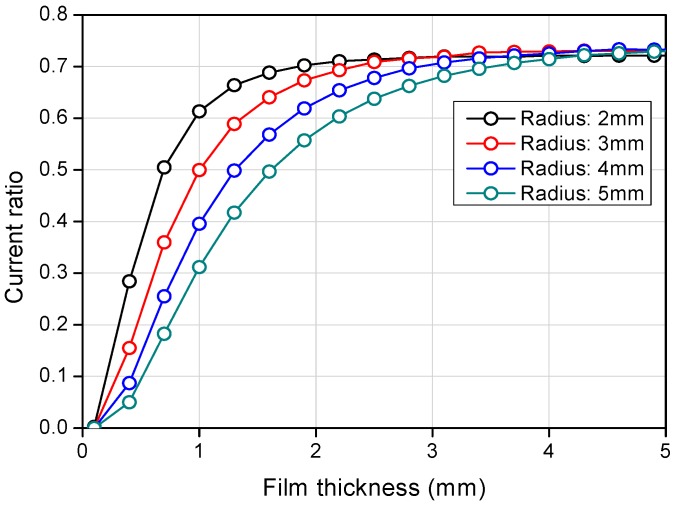
Electrical potential field calculation result according to the radius size.

**Figure 7:**
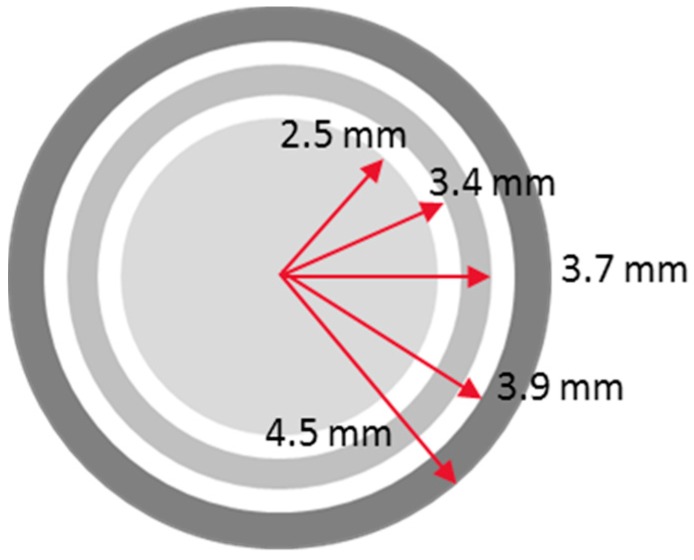
Specific dimensions of the electrodes.

Figure 5a shows the calculation domain of the COMSOL simulation. A plane water film with electric conductivity covered the ring-type electrodes, and the other boundary of the bottom plane was set to simulate an insulated plane. Electric potential of 1V was applied to the transmitter electrodes, with the receivers set to the ground potential. The electrical potential field was developed, as presented in Figure 5b, and the electric flux diffused from the center transmitter and converged to the two receivers. The predicted value of the current ratio depending on the liquid film thickness is indicated in Figure 6 with various radii of the outmost ring ($R_{r1}$). The current ratio increases with the liquid film thickness and becomes saturated to a certain value as the liquid film becomes thicker. According to the calculation, a larger outmost diameter of the sensor element produces better characteristics when measuring the target liquid film thickness. Contrary to this, a large sensor element reduces the degree of integration of the sensor array. For this reason, a radius of 4.5 mm and a pitch of 15 mm were selected for the present sensor. Afterwards, a series of calculations were conducted to determine the specific dimensions of the electrodes. According to numerical simulations, increasing the outer ring width increases the slope of the current ratio along with the liquid film thickness, but the signal becomes saturated at thinner thicknesses if the width is too large. In contrast, increasing the inner ring width increases the saturation thickness but makes the current ratio signal insensitive. Thus, the prototype sensor design was determined considering these two effects and restrictions during the manufacturing step. Finally, the dimensions of the ring-type electrodes were determined, as presented in Figure 7.

As crosstalk distorts the signal of the sensor, a ground electrode is required to absorb the leakage current. In this study, the sensor elements were uniformly distributed with a 15 mm pitch, and a ground electrode was added between the elements, as shown in Figure 4. The ground electrode had a lattice shape with a narrow width. In order to determine the width of the ground electrode, an additional electric potential calculation was conducted, indicating that a 0.2 mm width of ground electrode could prevent crosstalk without lowering the sensitivity during thickness measurements. Compared to the sensor design by Kim et al. (Figure 3), the proposed ring-type sensor has slightly lower sensitivity and a smaller measurement range. This arises due to the integration of the sensor elements in a confined region. Contrary to the sensor design by Kim et al., the proposed sensor is designed for multi-point measurements. Due to the reduced sensor element size, the signal sensitivity with regard to the thickness of the liquid film was reduced to prevent signal interference (crosstalk) caused by neighboring elements. Although electrical interference was unavoidable in this study, reasonable sensitivity and measurement ranges were achieved with a proper ground electrode design.

### 2.3. Fabrication of a Prototypic Sensor and Data Acquisition Setup

**Figure 8:**
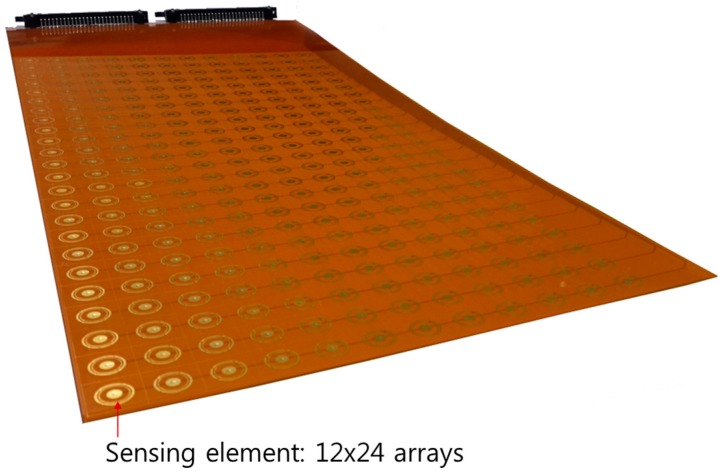
Configuration of the prototype sensor.

**Figure 9:**
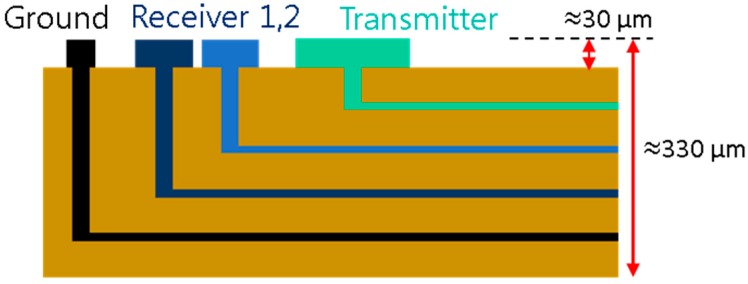
Cross-sectional view of the FPCB.

Based on the electrode design as determined by electrical potential calculations, a prototype sensor was fabricated on an FPCB with a 24 × 12 array of the sensing element, as shown in Figure 8. The dimension of the measurement part is 360 mm × 180 mm (height and width, respectively). A cross-sectional view of the prototype sensor is presented in Figure 9. The substrate material of the FPCB is polyimide and the thickness of each layer is approximately 13 μm, with electrode and circuit wire consisting of copper with a thickness of 18 μm. The electrode has an initial layer of nickel on the copper surface, followed by a thin protective layer of gold. The total thickness of the FPCB sensor is approximately 330 μm, and there are grooves (30 μm) between the electrodes and polyimide film. As the FPCB is composed of four layers, each group of signal lines was separated to prevent a short circuit.

**Figure 10:**
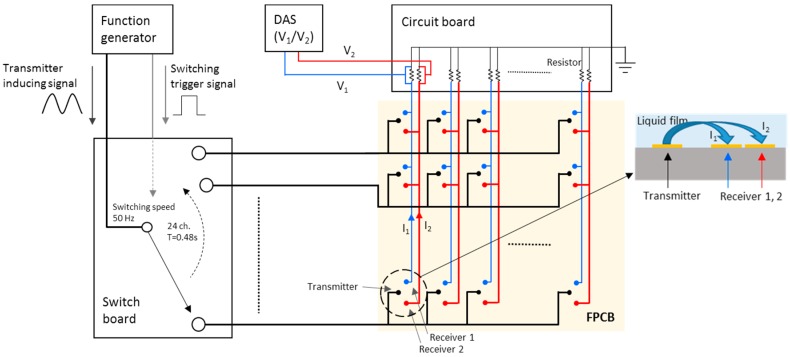
Signal transfer system with the FPCB sensor.

Figure 10 shows the overall signal processing unit of the FPCB sensor. Twelve transmitter electrodes located in the same row are connected to each other, thus giving the sensor 24 transmitting lines. Then, using a switching device, a single transmitting line is activated, moving electrically from the top to the bottom to cover all 24 transmitting lines. In addition, 24 receiver-1 electrodes and 24 receiver-2 electrodes positioned in the same column are connected in parallel; the sensor in this case has 12 receiver-1 lines and 12 receiver-2 lines. The input voltage was imposed by a function generator, and an AC sine wave was used to transmit the signal. The receiver lines are connected to a converter circuit board where the output currents are converted to voltage signals. These voltage signals are then transferred to a data acquisition system. As presented in Figure 10, the switching device changes the channel of the transmitter line such that the measurements of the liquid film thickness are conducted sequentially. Channel switching can be conducted both manually and automatically. For automatic switching, the switching board changes the channel using a trigger signal from the function generator. In this experiment, the frequency of the switching trigger signal is confined to 50 Hz due to a performance limitation of the data acquisition module. At this frequency, it takes about 0.5 s to switch all 24 transmitting lines, and the entire sensor domain can be accounted for by approximately 2 Hz. The data acquisition system has 24 channels of receivers, and each channel receives data at a sampling rate of 500 Hz.

### 2.4. Calibration of the Prototypic Sensor

**Figure 11:**
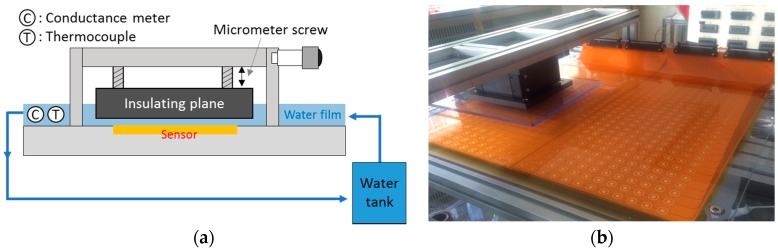
(**a**) Schematic diagram; (**b**) setup of the calibration device.

**Figure 12:**
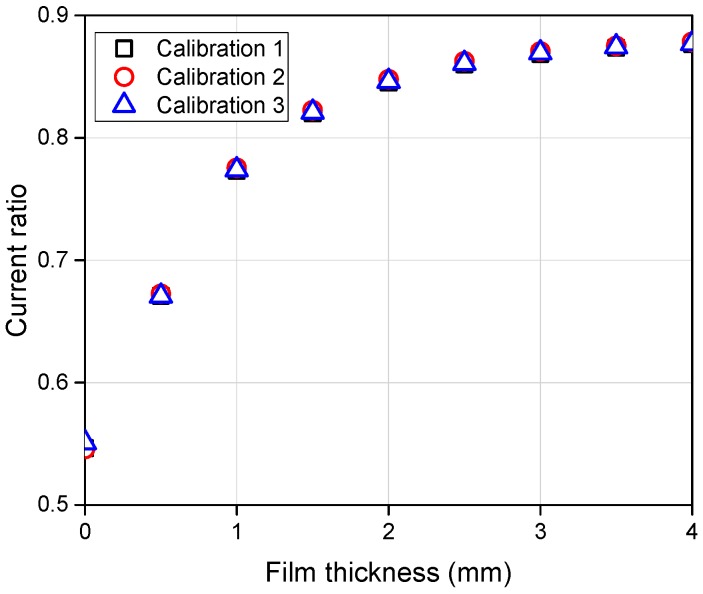
Calibration result (repeatability test).

**Figure 13:**
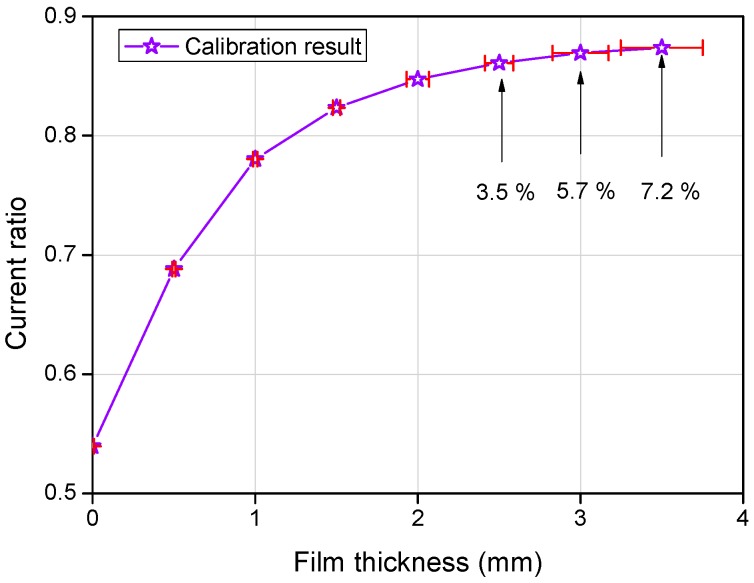
Calibration curve with spline interpolation.

Because every probe composing the FPCB sensor has different characteristics with regard to the others owing to fabrication tolerance, calibration should cover all probes for application to liquid film flow measurements. Given that the size of the modified FPCB sensor is too large for calibration in one trial, calibration was conducted by dividing it into four sub-sections. A schematic diagram of the calibration device and its setup are presented in Figure 11. The calibration range was 0–3.0 mm with 0.5 mm steps and the water condition for calibration was 18 °C and 22 μS/cm. Figure 12 illustrates a typical example of the calibration process after three repeated runs. With an increase in the liquid film thickness, the current ratio increases and then becomes saturated at a thickness of 3.5 mm. The offset point at a film thickness of zero is considered to be caused by the thin remaining liquid layer when the insulating plane of the calibration device makes contact with the sensor or the high impedance of the FPCB due to the complex and integrated circuit. Even if this result does not affect the measurement significantly, reducing the impedance of the circuitry is desired to improve the resolution of the sensor. A calibration curve was derived, as shown in Figure 13; this is a spline interpolation curve based on the calibration result. This analysis showed that for film thicknesses up to 1.5 mm, absolute accuracy of 0.025 mm is achieved with a relative average error of 1.6%. For film thicknesses up to 3.0 mm, the error increases to 0.25 mm and the maximum relative error is 5.7 % in a film thickness range of 0.0–3.0 mm. These values are considered as the bias error of the sensing element. This is a typical calibration result with a single sensing element, and the same procedure was repeated for all 288 sensing elements to produce calibration curves for all elements. In addition, a comparison with an ultrasonic thickness gauge was conducted with the prototype sensor to ensure of the reliability of the sensor. The maximum error between the two methods was 5.3% and the average error was 1.8% [11].

**Figure 14:**
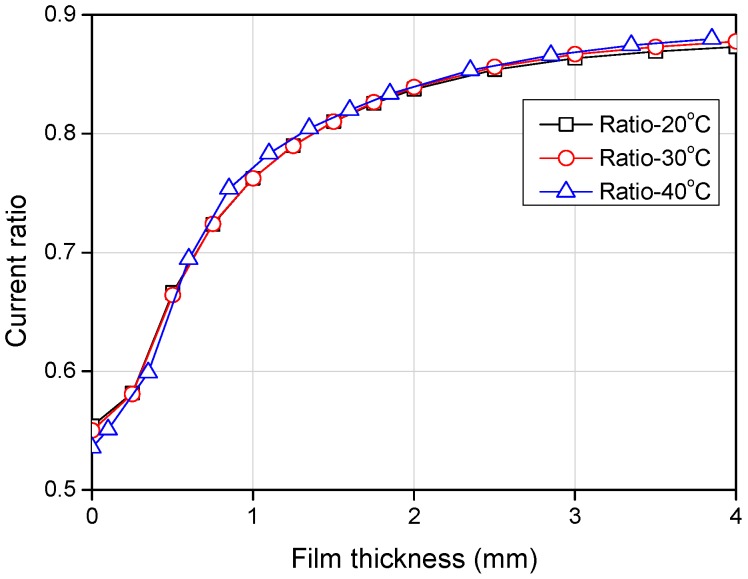
Current ratio with different temperature conditions.

Afterwards, the characteristics of the sensing element at three different temperature conditions were investigated and the output current ratios were plotted with the film thickness (Figure 14). This graph shows that the current ratios obtained in the range of 20 °C–40 °C were in fairly good agreement with the others with an average error of 3.9%. However, if the temperature difference exceeds this value, a distorted signal results and the current ratio shows dependency on the fluid temperature, in contrast to the theoretical analytical results. The reason behind this distortion remains not fully understood. One possible reason would be the different impedance changes between the two receiver channels with respect to the water temperature. Slight distortion of the acrylic plates in the calibration device onto which the sensor was attached and how the calibration was done can also cause this unexpected result. Not only for this particular sensing element but also for most other elements, a similar magnitude of error results if the temperature difference exceeds 20 °C. This imposes a limitation on the application range of the present sensor, and this should be improved for a wider range of applications in the future.

To sum up the performance of the sensor, the three-electrode sensor has a measurement range of 0.0 mm–3.0 mm. Moreover, it has 1.6% error up to 1.5 mm and 5.7% up to 3.0 mm. With regard to temperature stability, 3.9% error was achieved in the temperature range of 20 °C–40 °C.

## 3. Feasibility Test Result of the Liquid Film Sensor

**Figure 15:**
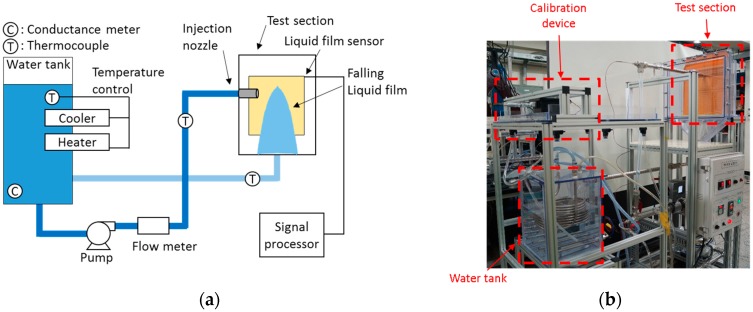
(**a**) Schematic diagram; and (**b**) configuration of the feasibility test.

**Figure 16:**
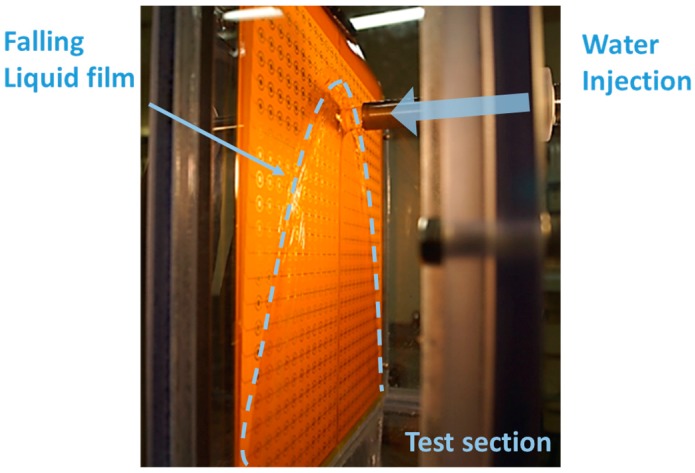
Edge of falling liquid film.

The calibrated sensor was applied for the thickness measurement of a falling liquid film after impinging on a plate wall. The apparatus for this feasibility test is illustrated and displayed in Figure 15a,b. The water was supplied by a pump, and its flow rate and temperature were measured by an electromagnetic flow meter and a k-type thermocouple, respectively, installed downstream of the pump. The water was injected from the nozzle onto a vertical wall where the FPCB sensor was attached. The distance from the nozzle to the FPCB sensor was 25 mm and the diameter of the nozzle was 21 mm. These geometries are identical to those in Yang et al. [7]. The impinged liquid flowed down along the sensor surface, making a shaped liquid film, as shown in Figure 16. The figure also shows how the sensor was installed and applied to the test section. Subsequently, the falling water exited the test section through the drain and flowed into a storage tank. In the storage tank, a heater and a cooler were installed to control the water temperature. An electrical conductance meter was also installed in the storage tank to monitor the water conductance. The details of the measurement instrument are summarized in Table 1.

**Figure 17:**
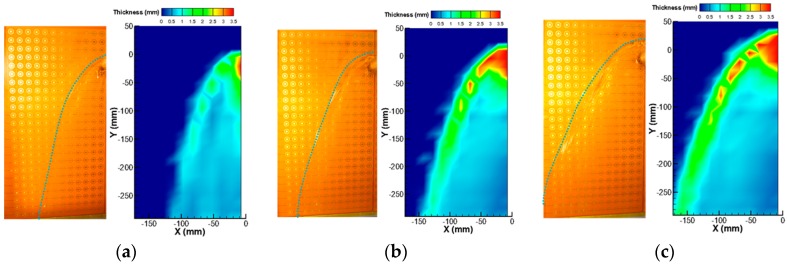
Local liquid film thickness of different liquid inlet velocities: (**a**) V_in_ = 0.48 m/s; (**b**) V_in_ = 0.68 m/s; and (**c**) V_in_ = 0.87 m/s.

Initially, the local film thickness was measured by the FPCB sensor while controlling the velocity of the water injection, with the water temperature constant at 20 °C. The water velocities at the nozzle were 0.48, 0.68, and 0.87 m/s, which cover the reference velocity condition in the experiment by Yang et al., i.e., 0.68 m/s. To determine the time-averaged thickness of the liquid film, and the measurement at each channel was continued for five seconds. Figure 17 shows the results of the local liquid film thickness measurement with three different liquid inlet velocities. The images on the left side are those of the actual liquid film observed by the camera, and dotted lines were added to indicate the edge of the film. The figures on the right depict the distributions of the local film thickness as measured by the present liquid film sensor, and the displayed contours are the time-averaged thicknesses. In this figure, the point at (0, 0) is the center point of the water injection nozzle.

Liquid film characteristics similar to those of the visual observation results were successfully reproduced in the film thickness measurement results. Initially, the thickest liquid film was measured near the center position where the liquid jet impingement occurs. In this region, the film thickness exceeded the measurement range of the present sensor; therefore, it had constant values of the maximum measurable thickness at 3.5 mm. The parabolic falling liquid film was reasonably reproduced by the present sensor. Near the liquid film edge, a thick film thickness was measured, consistent with the visual observation due to the surface tension force exerted on the edge of the liquid film. Oscillating edge behaviors were also observed in the visualization, and, intermittently, droplets were generated and became reattached to the wall. Owing to this oscillation and entrainment near the edge, an irregular liquid film thickness appeared near the film edge. In general, the liquid film width increased with the liquid injection velocity, and thicker film was measured with it, especially near the edge. From these measurement results and in comparison with the visual observations, it was found that the present sensor could measure liquid film thicknesses reliably under constant water temperature conditions.

**Figure 18:**
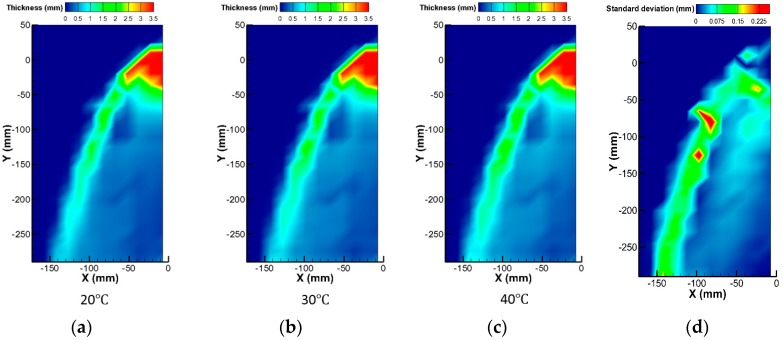
Liquid film thickness with different temperature conditions: (**a**) 20 °C; (**b**) 30 °C; (**c**) 40 °C; and (**d**) standard deviation.

A feasibility test to evaluate the working performance of the sensor under varying temperature conditions was then conducted. The liquid injection velocity was 0.63 m/s and the water temperature was changed from 20 °C to 40 °C in 10 °C steps. Figure 18 shows the measured liquid film thickness with three different water temperature conditions. Even with these temperature differences, a comparable liquid film thickness distribution was obtained. Figure 18d shows the standard deviation distributions of the liquid film thickness in these three measurements. Owing to the oscillating nature of the liquid film edge, the liquid film thickness deviation was significant at the liquid film edge compared to the other regions. In particular, the location of the liquid entrainment from the film edge varied slightly depending on the experimental conditions. Consequently, the largest deviation between the measured data was observed at the positions where droplet entrainment occurred. Except for these regions, the deviation was less than approximately 0.07 mm, and the temperature compensation capability of the three-electrode liquid film sensor was confirmed with this mild temperature variation.

## 4. Conclusions

In order to confirm the feasibility of the three-electrode conductance method for liquid film thickness measurements, which is expected to be advantageous under varying temperature conditions, two prototype liquid film sensors were fabricated on an FPCB. The designs of the prototypic sensors were determined by electrical potential analyses. The calibration tests of these sensors showed that the fabricated sensors are applicable in conditions with mild temperature variances at temperatures lower than 20 °C. Subsequently, the liquid film sensor was applied to a plate duct and to a condition with a falling liquid film thickness with impingement on a wall and was measured with a 12 × 24 sensing array. The measurement results showed reasonable consistency with the visual observations, and the feasibility of the three-electrode conductance sensor was confirmed in the temperature range of 20 °C–40 °C.

However, the possible temperature variation range is limited to 20 °C in the present design and experimental setup. In order to overcome this limitation, an improvement of the calibration devices is initially required. Additional investigations are then needed to determine the cause of the unexpected temperature dependency of the current ratio using a counterpart liquid film thickness measurement sensor. Reducing the impedance of its circuitry is required as well for more accurate resolutions by enlarging the signal-to-noise ratio.

## Figures and Tables

**Table 1:** Span and accuracy of the measurement instrument.

Instruments (Model)	Span	Accuracy
Thermocouple (K-type)	−200~1250 °C	±2.2 °C
Electrical conductance meter (EUTECH Instruments CON450)	0~200 mS/cm	0.01 μS/cm
Flow meter (Toshiba GF630)	0.0~10.0 m/s	±0.2%
